# Magic bullets, magic shields, and antimicrobials in between

**DOI:** 10.1016/j.pscia.2022.100002

**Published:** 2022-12-13

**Authors:** Praveen Prathapan

**Affiliations:** New Biochemistry, University of Oxford, South Parks Road, Oxford, OX1 3QU, UK

**Keywords:** Immunomodulatory antimicrobial, Pan-pathogen pharmacology, Pandemic preparedness

## Abstract

There are only two classes of small-molecule drugs for infectious disease: pathogen-directed antimicrobials and host-directed immunomodulators. The former includes antibiotics and antivirals while the latter comprises corticosteroids such as dexamethasone. Here I inaugurate a third class, immunomodulatory antimicrobials (IAs), which considers small-molecule drugs harbouring both pathogen-directed and host-directed pharmacology. I review seven types of IAs, and argue that their high repositionability and network pharmacological ability to counter multiple pathogen types render them more applicable to pandemic-preparedness research than antivirals.

## 1 Introduction

It is said, that on his deathbed, Louis Pasteur retracted his germ theory of disease. Conceding, ‘*Le microbe n’est rien, le terrain est tout*’ (The microbe is nothing, the terrain is everything), Pasteur had spent decades propagating the idea that pathogens are the cause of infectious disease [1]. In contrast, the terrain theory (or host theory), championed by Pasteur's contemporaries Claude Bernard and Antione Béchamp, contended that an unhealthy bodily environment causes disease [2].

In the 20th century, Paul Ehrlich's ‘magic bullet’ concept complemented Pasteur's work by defining a treatment development paradigm for infectious diseases based upon using small-molecule drugs to kill pathogens [3]. From the discovery of penicillin in the 1920s to the development of antivirals in recent decades, the magic bullet paradigm is a century-spanning success in combatting the plethora of pathogens that cause disease [4,5]. Contemporary antimicrobial nomenclature still reflects this pathogen-centred approach: the term ‘antibiotic’ derives from the Greek αντι *anti*, ‘against’ and βíoc *bios*, ‘life’ - literally ‘opposing life’ [6].

The most well-known limitation of the magic bullet approach to antimicrobial development is the concomitant rise of antimicrobial resistance (AMR) over the last century, which has occurred in all pathogen classes [7]. The second limitation has only come to light recently, when the 2020 pandemic saw the emergence of a novel infectious disease unabated by existing pharmaceuticals [8]. While it was hoped that antiviral drugs such as remdesivir would be effective against the novel disease, instead the small-molecule corticosteroid dexamethasone arose as a successful host-directed approach to treat severe COVID-19: the first ‘magic shield’ [9].

The need to develop immunomodulators to prepare against novel infectious diseases is not a new argument: in 1987 Stanley Wiener envisioned the use of ‘broad-spectrum… agents that enhance the immune system [to] provide a non-specific, but effective, medical defense in the future’ [10]; in 2007, the Strategic Plan for Biodefense Research by the U.S. Department of Health and Human Services described the need for general emergency pills for global health crises [11]. By targeting the host immune system instead of a specific pathogen, therapeutics could indeed become ‘non-specific’ insofar as being deployable against a range of pathogens. The broader the pathogen range of a therapeutic, the more suitable for ‘Pathogen X’, a hypothetical, unknown pathogen that could cause a future epidemic. Likewise, ‘Drug X’, a hypothetical therapeutic that is repositionable against the widest range of pathogen types, must harbour an equally broad pharmacological profile in anticipation of novel diseases.

I have discovered two classes of host-modulating drugs that display efficacy against multiple pathogen classes, a pharmacological property hereby termed ‘pan-pathogen pharmacology’ (PPP). The first is small-molecule immunomodulators such as dexamethasone, whose success in treating both bacterial infections as well as COVID-19 underscores the notion that immunomodulators can treat infectious disease across pathogen classes [12]. The second is pathogen-inhibiting antimicrobials which also exhibit immunomodulatory activity, henceforth termed ‘immunomodulatory antimicrobials’ (IAs) [13]. As with typical antimicrobials and immunomodulators, IAs shift the damage-response curve towards reducing damage to the host [14]. However, they exhibit both pathogen-directed and host-modulating capabilities: a polypharmacological property which bestows their propensity to treat disease across pathogen classes [15].

This review contends that the magic shield must supersede the magic bullet as the goal of antimicrobial development. While recognition of the magic shield paradigm confutes Ehrlich's magic bullet, it addresses AMR and COVID-19, reinstitutes Bernard and Béchamp's terrain theory, and unlocks the possibility of developing a single drug for all infectious disease, both extant and emergent [16].

## 2 IAs

**Fig. 1:**
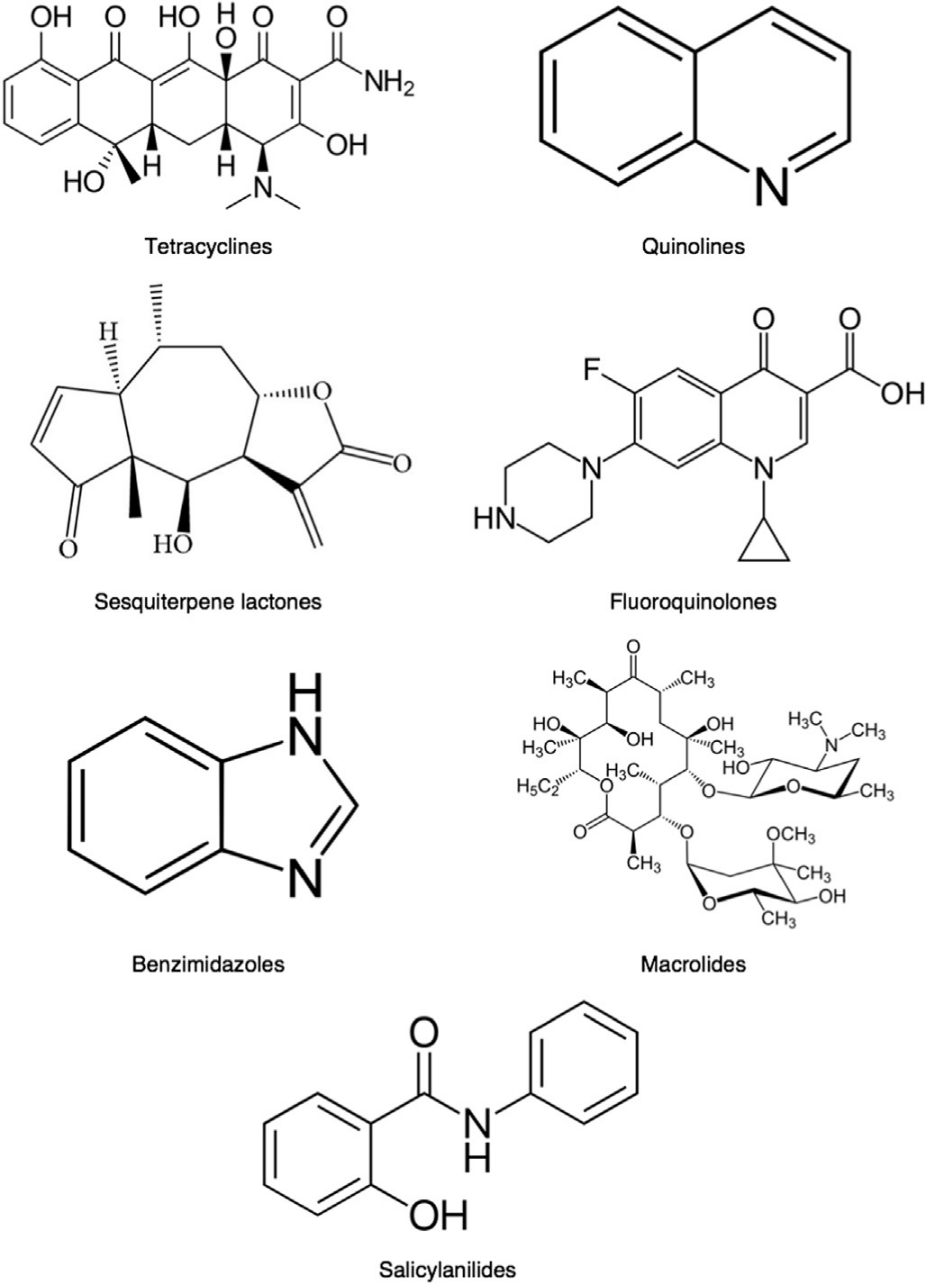
**Seven classes of IAs.** IAs comprise a heterogeneous subset of compounds which display both pathogen-killing (magic bullet) and host-directed (magic shield) properties. This hallmark is achieved by polypharmacological perturbation of signalling networks within the host-pathogen interactome (network pharmacology).

Although both IAs and magic shields exhibit PPP, the PPP for IAs is more assiduously characterised. Magic shields like dexamethasone, while useful for treating bacterial meningitis and COVID-19, have long been known to exacerbate symptoms when used to treat other infection types [17]. Instead, the unique bullet/shield network pharmacology of IAs appears to underscore their applicability for a broader range of communicable diseases. The pharmacology of seven classes of IAs is henceforth discussed: tetracyclines, quinolines, sesquiterpene lactones, fluoroquinolones, benzimidazoles, macrolides, and salicylanilides (Fig. 1).

### 2.1 Tetracyclines

The first IA class comprises the oldest antibiotics. Discovered in the 1940s, tetracyclines are the archetypal antibiotics that have been repositioned against a variety of disease types from malaria to cancer, all while their magic shield pharmacological profiles are ascertained [[18], [19], [20], [21], [22], [23], [24]].

The magic bullet properties of tetracyclines are well characterised [25]. They inhibit protein synthesis of bacteria by binding reversibly to the bacterial 30 ​S ribosomal subunit and preventing the aminoacyl tRNA from binding to the A site of the ribosome [26]. They also partially bind the bacterial 50 ​S ribosomal subunit and may alter the cytoplasmic membrane to cause intracellular components to leak from bacterial cells [27,28]. With regard to malarial infection, tetracyclines block expression of the apicoplast genome, driving distribution of non-functional apicoplasts into daughter merozoites and leading to a potent antimalarial effect [29,30].

The development of immunomodulatory tetracyclines, such as doxycycline and minocycline, is a breakthrough in antibiotic therapy and recent investigations have shed light on their mechanisms of action [31,32]. Both potentiate the innate immune response and incite resolution of inflammation [33,34]. Immunomodulatory tetracyclines were recently shown to potentiate macrophage cytokine release *in vitro*, and while improving mucosal recovery in colitic mice, minocycline enhanced pro-inflammatory cytokine release, including IL-1β, IL-6, and IL-4, in contrast to macrolides which dampen the cytokine storm. However, there is also evidence of the ability of minocycline to reduce pro-inflammatory cytokine release in non-communicable diseases, warranting further investigations [35].

Overall, the magic shield properties of tetracyclines, manifesting as a plethora of mechanisms modulating host immune pathways, reposition this drug class to an equally broad range of disease states [[36], [37], [38], [39], [40], [41]]. Indeed, the study of tetracyclines as antivirals is over 50 years old, with the first available report on their antiviral activity being published by Negrette et al. in the 1960s [42]. More recently, a range of tetracycline antibiotics were inducted into clinical trials for COVID-19 due to their ability to chelate and inhibit host matrix metalloproteinases (MMPs) which are co-opted by RNA viruses [[43], [44], [45], [46], [47], [48], [49]]. Lastly, a combination of tetracycline and a macrolide (another IA class) has been suggested as a possible treatment regimen for COVID-19, intimating the potential benefits of combinatorial IA treatment [50].

### 2.2 Quinolines

Comparable with the clinical history of tetracyclines for bacterial diseases is the clinical history of quinolines for malaria. A heterocyclic aromatic organic compound, quinoline is characterised by a double-ring structure containing a benzene ring fused to pyridine at two adjacent carbon atoms [51]. While quinoline itself has few applications, its derivatives exhibit broad antimalarial and anticancer activity [52,53].

Quinolines are magic bullets against bacteria, malaria, and fungi. Nitroxoline (8-hydroxy-5-nitroquinoline) is an 8-hydroxyquinoline derivative that has been prescribed as an antibiotic for decades in Europe to treat urinary tract infections, its antibiotic activity being attributed to its ability to chelate metal ions (akin to tetracyclines) and to dispel biofilms [54]. Chloroquine is a protonated, weakly basic 4-aminoquinoline that accumulates in the food vacuole of parasites and interferes with degradation of host red blood cell (RBC) haemoglobin, preventing malarial parasite growth [[55], [56], [57]]. Unmodified quinoline exhibits relatively high activity against some fungal strains at non-toxic concentrations and the fungistatic activity of 8-hydroxyquinoline and its metal complexes has been known since the early 1920s, with these compounds now broadly used in healthcare [58].

2020 saw many reviews highlighting the immunomodulatory activities of chloroquine and hydroxychloroquine [59,60]. At concentrations less than 20 ​μM, both inhibit activation of nucleic acid sensors, Toll-like receptors (TLRs) in endosomes, and cyclic GMP-AMP synthase (cGAS) in the cytoplasm [61]. This leads to inhibition of pattern recognition receptor (PRR)-induced activation of downstream pro-inflammatory cytokine and type I interferon (IFN) gene expression [62]. At concentrations higher than 100 ​μM, chloroquine and hydroxychloroquine increase lysosomal pH, leading to disruption of presentation of extracellular antigens processed through the endolysosomal pathway and intracellular antigens processed through the autophagosome-lysosome fusion pathway by antigen presenting cells [63,64]; this property, revisited in Section 3, is a prospective hallmark of IAs. The extensive magic shield pharmacology of quinolines has likely contributed to their use as antiproliferative agents [[65], [66], [67], [68], [69], [70], [71], [72], [73], [74]]. Overall, recognition that quinolines are IAs affirms the untapped repositioning potential of these antiparasitic agents for cancer, viral pandemics, and bacterial and fungal infection [[75], [76], [77], [78], [79], [80], [81]].

### 2.3 Sesquiterpene lactones

As chloroquine was first extracted from the bark of *Cinchona officinalis* trees, so sesquiterpene lactones have been extracted from *Artemisia annua* wormwood plants. Sesquiterpenes are a class of terpenes that consist of three isoprene units; sesquiterpene lactones are a class of sesquiterpenes that contain a lactone ring [82]. Artemisinin, a new, highly effective antimalarial compound, is a sesquiterpene lactone found in *Artemisia annua* [83]. It was discovered in 1972 by Tu Youyou, who shared the 2015 Nobel Prize in Physiology or Medicine for its discovery [84].

Artemisinin-based combination therapies (ACTs) are considered standard treatment worldwide for *P. falciparum* malaria as well as malaria due to other species of *Plasmodium* [85]. Artemisinin's mechanism of action includes induction of oxidative stress in infected RBCs [86]. However, evidence of artemisinin resistance was discovered in several regions in Southeast Asia, emphasising the need for alternative antimalarial medications such as doxorubicin, a potential IA [87]. Artemisinin and its derivatives exhibit antimicrobial activity against Gram-positive bacteria, Gram-negative bacteria, including *Mycobacterium tuberculosis*, *Staphylococcus aureus*, *Escherichia coli*, and *Helicobacter pylori* [88]. Artemisinin also displays an inhibitory effect on hepatitis C virus JFH-1 [89]. Last year, it was reported that artemisinin-based treatments display efficacy against SARS-CoV-2 *in vitro*, a contemporary demonstration of the potential of sesquiterpene lactones, and natural products in general, for pandemics [90].

The immunomodulatory activities of artemisinin and its derivatives on autoimmune diseases have been reviewed recently [91]. Sesquiterpene lactones have the capacity to regulate expressions of pro-inflammatory and anti-inflammatory cytokines, frequency and activation of T helper and B cells, and responsiveness of macrophages, DCs, neutrophils, mast cells, and MDSCs [92]. Consequently, artemisinin and its derivatives have a diverse application portfolio ranging from systemic lupus erythematosus (SLE) and multiple sclerosis to transplant rejection and cancer [[93], [94], [95], [96], [97], [98], [99], [100]]. The recent discovery of sesquiterpene lactones likely prefaces the characterisation of a broad-spectrum pharmacological profile in forthcoming years.

### 2.4 Fluoroquinolones

There is growing appreciation for the synergistic effect of artemisinin with fluoroquinolones for the treatment of malaria. Quinolone antibiotics are broad-spectrum bactericides that share a bicyclic core structure related to the substance 4-quinolone; addition of a fluorine atom at position C-6 has yielded fluoroquinolones, such as ciprofloxacin and moxifloxacin [101,102]. Today, nearly all quinolone antibiotics in use are fluoroquinolones, and are used in human and veterinary medicine to treat bacterial infections, as well as in animal husbandry, particularly poultry production [103]. It has been understood since the 1990s that fluoroquinolones used for the treatment of bacterial infections exert activity against fungi, parasites, and viruses [[104], [105], [106]]. That fluoroquinolones have been recommended for polymicrobial infections is simultaneously the recognition of the PPP of fluoroquinolones and a symptom of pre-COVID-19 thinking; the antimicrobial range of fluoroquinolones harbours significant untapped potential for pandemic-preparedness and bioterrorism-preparedness research i.e., preparing for a ‘monomicrobial’ disease by an unknown pathogen [107].

A demonstration of the heterogeneity of PPP, fluoroquinolones act as magic bullets against bacteria and parasites. With the former, fluoroquinolones inhibit two DNA topoisomerases essential for bacterial DNA synthesis and replication. Likewise, gyrase, a prokaryotic type II topoisomerase that serves the apicoplast genome (plDNA) of *Plasmodium falciparum*, is poisoned by fluoroquinolones which stabilise a catalytically inert ternary complex of enzyme, the plDNA substrate, and inhibitor; a mechanism perhaps responsible for the commercialisation of over 20 quinolones for malaria [108,109]. Recently, several clinafloxacin-triazole hybrids presented antifungal activity against *Candida albicans* and *Candida mycoderma* [110]. Finally, an *in silico* study in 2020 showed that fluoroquinolones ciprofloxacin and moxifloxacin may inhibit SARS-CoV-2 replication by exhibiting stronger capacity for binding to its main protease than chloroquine and nelfinavir, a protease inhibitor antiretroviral drug [111].

Fluoroquinolones display multiple magic shield immunomodulatory actions leading to attenuation of the inflammatory response via inhibition of pro-inflammatory cytokines, albeit by unknown mechanisms and signal transduction pathways [112,113]. It has also been demonstrated that, in analogy to chloroquine and tetracyclines, fluoroquinolones bind to and insert into cellular membranes, altering their fluidity [28,114]. It would be interesting to compare the *in vitro* physicochemical interactions of these compounds with the lysosomotropic properties of the macrolide azithromycin, a broad-spectrum therapeutic. Indeed, both have been hypothesised to exert global physiological changes by altering pH [115,116]. In addition to their use as first-line therapeutic agents for the management of severe community-acquired pneumonia, their favourable pharmacokinetics and safety profiles, and higher concentrations in the lungs in the case of levofloxacin and moxifloxacin, it has been proposed that respiratory fluoroquinolones be used in the treatment of SARS-CoV-2-associated pneumonia, furnishing this IA class with both magic bullet and magic shield pharmacology against COVID-19 [[117], [118], [119]]. It is likely that the future of the fluoroquinolone class of IAs will comprise synthesis of an increasing array of hybrids and modifications, which in turn will reinforce the applicability of fluoroquinolones and their derivatives to many fields of medicine [[120], [121], [122], [123], [124], [125], [126], [127], [128], [129], [130], [131], [132], [133]].

### 2.5 Benzimidazoles

To combat AMR, many fluoroquinolones have been hybridised with benzimidazoles. Benzimidazole is a heterocyclic aromatic organic compound composed of benzene and imidazole [134]. The properties of benzimidazole and its derivatives have been studied for over a century and substituted benzimidazole derivatives have found applications in diverse therapeutic areas such as antiulcers, anticancer agents, and anthelmintics.

Antiparasitic effects of benzimidazoles are achieved by polypharmacological interference with sugar metabolism, alteration of energetic processes, and binding to tubulin to affect the cell cycle [135]. Many anthelmintic drugs such as albendazole, mebendazole, and triclabendazole also belong to the benzimidazole class of compounds [136]. Benzimidazole fungicides are commercialised, deriving their success by their magic bullet ability to bind fungal microtubules to inhibit hyphal growth [137]. Benzimidazoles also bind spindle microtubules to block nuclear division [138]. These cellular processes are also compromised in cancer and recent studies have indeed shown that benzimidazole derivatives exhibit prominent antineoplastic activity, further attesting to how the broad repositionability of IAs can also derive from their magic bullet properties [139]. Indeed, applications of benzimidazole-based compounds range from proton-pump inhibitors (antacids) to nitazenes (benzimidazole opioids). In the wake of COVID-19, benzimidazole-pyrazole hybrids are being synthesised with a view to address the dearth of suitable antiviral drugs [140,141].

### 2.6 Macrolides

The quintessential antibiotics, it has long been accepted that macrolides exhibit a diverse range of properties with confirmed activity in prokaryotes and effects on inflammatory and immune cells, mucus secretion, and epithelial cell differentiation [142]. In fact, anti-inflammatory and immunomodulatory actions contribute to the therapeutic benefit of macrolides in most of the infectious disorders for which they are approved, as well as rationalising their application for other chronic inflammatory conditions that do not have primarily infectious aetiologies [143]. The chemical structures of certain macrolides are biomimetic, aiding their interactions with components of mammalian cells [144].

Macrolides constitute some of the most well-characterised magic bullets of the 20th century, including one of the most consequential antibiotics: erythromycin, which inhibits protein synthesis by binding 23 ​S rRNA in the 50 ​S subunit of ribosomes in susceptible bacteria [145]. Azithromycin, an erythromycin derivative, inhibits protein synthesis in the apicoplast of *Plasmodium falciparum* whilst blocking invasion of RBCs [146]. The antiparasitic ivermectin has been christened a ‘wonder drug’ due to its PPP [147]. Macrolides have been used in the treatment of HIV, herpes simplex type I, and influenza though this likely involves magic shield host-directed mechanisms [144]. That being said, amphotericin B boasts a feature on the WHO's *List of Essential Medicines* for its ability to ameliorate systemic fungal infections; its magic bullet mechanism involves binding ergosterol leading to formation of pores in the fungal cell membrane, ion leakage, and ultimately fungal cell death [148].

The myriad magic shield properties of macrolides are still being elucidated. Atopic dermatitis and organ transplant rejection are addressed by immunosuppressant macrolide administration [149,150]. Azithromycin-induced macrophage polarisation appears associated with alteration of STAT1 signalling through inhibition of NF-κB mediators [[151], [152], [153], [154]]. An improved understanding of the mechanisms associated with these agents could lead to promising therapeutic target discovery in a plethora of communicable and non-communicable immune disorders.

From the treatment of viral, bacterial, and fungal infections to the near-eradication of onchocerciasis, to supporting successful organ transplants, and to mitigating cancer, the applications of this IA class have had a profound impact on human health [[155], [156], [157], [158], [159], [160], [161], [162], [163], [164], [165], [166], [167], [168]]. My own discovery of IAs began with consolidating the PPP of azithromycin in 2020 during the first wave of the COVID-19 pandemic, and recent quantitative structure activity relationship (QSAR) studies provide new *in* silico and *in vitro* data pertaining to the ability of macrolides to impede entrance of SARS-CoV-2 into host cells [169,170]. Macrolides are the prototypical IA class and as such buttress a benchmark against which other IA classes may be assessed.

### 2.7 Salicylanilides

Anthelmintic salicylanilides are amides of salicylic acid and aniline [171]. Owing their discovery to research for new antiseptics, salicylanilides have a variety of pharmacological uses: chlorinated derivatives, including niclosamide, oxyclozanide, and rafoxanide, are used as anthelmintics, especially as flukicides; and brominated derivatives, including dibromsalan, metabromsalan, and tribromsalan, are used as disinfectants with antibacterial and antifungal activities [172]. In a sharp albeit dangerous demonstration of the power of ideas, former President Trump stated ‘I see the disinfectant that knocks it out in a minute … is there a way we can do something like that by injection inside or almost a cleaning? It would be interesting to check that.’ [173,174] By giving rise to both intravenous antimicrobials and surface disinfectants, the broad-range chemistry of salicylanilides is unironically a critical avenue for pandemic-preparedness research [175,176].

Niclosamide and nitazoxanide are notable salicylanilides with clinical appraisals for a range of infectious diseases. Though it is unclear how they exert their antibacterial effects, their magic bullet antiparasitic mechanisms have begun to be delineated. As a hydrogen ionophore, niclosamide translocates protons across the inner membrane of mitochondria, resulting in the uncoupling of oxidative phosphorylation from electron transport and inhibiting the production of ATP. These effects are observed in isolated mitochondria of helminths and mammals. Selective toxicity is achieved by minimal systemic absorption combined with protective influence of protein binding. Niclosamide, an anthelmintic agent used to treat parasitic infections, also exhibits broad-spectrum antibacterial and broad-spectrum antiviral properties *in vitro* [177]. Nitazoxanide is a nitrothiazole derivative of salicylamide and is synthesised using the scaffold of niclosamide [178]. Studies of protozoa and anaerobic bacteria have shown that nitazoxanide is a magic bullet inhibitor of pyruvate-ferredoxin oxidoreductase (PFOR), an enzyme essential to anaerobic energy metabolism. However, interference with the PFOR enzyme-depending electron transfer reaction may not be the only pathway by which nitazoxanide exhibits antiprotozoal activity, and the mechanism of nitazoxanide's activity against helminths is unknown.

It has been suggested that the broad activity of niclosamide is due to its magic shield immunomodulating activity [179,180]. In 2012, niclosamide was discovered to be an entry inhibitor for a number of pH-dependent respiratory viruses, including influenza virus and human rhinoviruses [181]. Cell-biological and biochemical analyses and intracellular pH measurements have revealed that niclosamide neutralises acidic membrane-bounded compartments. Furthermore, niclosamide blocks rhinovirus infections synergistically with the macrolide proton ATPase inhibitor bafilomycin A1 [182]. Physico-chemical interference of host pathways is thus a leitmotif in IA pharmacology.

Though it is primarily used as an antiparasitic agent, nitazoxanide has been described as a ‘first-in-class broad-spectrum antiviral agent’ [183]. Indeed, much of its PPP stems from its ability to perturb the host immune landscape. First and second generation thiazolides upregulate gene expression of multiple components of the TLR- and type I IFN-signal transduction pathways, of multiple chemokines and cytokines, as well as production of IFNα by pDC, and that of IL2 and IFNγ by CD4^+^ T lymphocytes [184]. A double-blind, placebo-controlled clinical trial of patients with influenza infection showed a clear effect of nitazoxanide in reducing the severity of flu symptoms and viremia, and in increasing seroprotection and seroconversion rates [185,186]. Moreover, immunomodulatory effects of thiazolides were stronger in FLU-stimulated cells compared to unstimulated control, suggesting that thiazolides reinforce host immune responses [187,188]. It has been noted that this remarkable selectivity of thiazolides seems to explain the lack of adverse clinical side effects of nitazoxanide [189]. Nitazoxanide's low cytotoxicity sets it apart from other immune-based therapies such as IFN, IL-2, and IL-12 that are associated with common and severe side effects resulting from generalised and extensive immune activation [190].

Due to their PPP, salicylanilides are preeminent IAs for pandemics, as evidenced by the ongoing campaign for their use against COVID-19 and monkeypox (also known as ‘mpox’) [[191], [192], [193], [194], [195], [196], [197], [198], [199]]. Of particular note is that their immunomodulatory properties are better characterised than their magic bullet pharmacology. A transdisciplinary link with detergent chemistry adds further intrigue to the seventh IA class.

## 3 IA properties

Previously, I described how common disease networks within the host interactome constitute a consistency in the innate immune response in the presence of differing classes of pathogens [200]. Similarly, confluent chemical and pharmacological properties of IAs likely explain their PPP; in particular cationic amphiphilicity and lysosomotropicity, which are interestingly also found in therapeutics with high repositioning potential, even outside of infectious disease.

### 3.1 Lysosomotropicity and cationic amphiphilicity

Lysosomotropicity describes the ability of a therapeutic to accumulate within lysosomes and involves the rapid vacuolisation of the intracellular microenvironment by weakly basic amine compounds. These compounds are characterised by a hydrophobic aromatic ring or ring system coupled with a hydrophilic sidechain containing an ionisable amine functional group and are known as cationic amphiphilic drugs (CADs). Lysosomotropic compounds, much like amphiphilic antimicrobial peptides (AMPs) are not only used as antidepressants, antihistamines, antimicrobials, and antipsychotics, but potentially selectively destroy cancer cells [201]. Indeed, lysosomotropicity has been described as a way of therapeutically maintaining cellular homeostasis for acute maladies and, particularly with regard to COVID-19, may bridge the gap between antiviral and anti-inflammatory pharmacology [202].

In accordance with their lipophilicity, CADs diffuse through the membrane of acidic organelles in their unionised form and become protonated in the acidic lumen. Due to their decreased membrane permeability, protonated molecules cannot cross back to the cytosol and get trapped inside the acidic lumen where they accumulate. This ion trapping results in an increase in lysosome volume and impairing of their function, leading to downregulation of autophagy, reduction of endocytosis, and phospholipidosis due to reduction of phospholipid degradation. Phospholipidosis is the phenomenon whereby various lipid species accumulate inside the late endosomes and lysosomes. This phenotype is akin to that of patients with Niemann Pick type-C (NPC) disease, a lipid storage disorder [203]. If not too extreme, all these effects can be reversed upon cessation of treatment.

Evidence supporting antiviral activity of CADs has been well reviewed [[204], [205], [206], [207]]. It has also been anticipated that many drugs registered for non-antimicrobial use, due to their lysosomotropicity, exhibit intrinsic antiviral effects and constitute a pool of potential existing antivirals that may prove useful as the first line of defence in new viral outbreaks [208]. Two antiviral CADs have already been described as achieving their efficacy by lysosomotropicity: tilorone and umifenovir. Though the last 50 years have seen the use of tilorone as an interferon-inducing broad-spectrum antiviral, it is currently registered and used only in Russia and Ukraine for indications such as influenza, acute respiratory viral infection, viral hepatitis, viral encephalitis, and myelitis [209]. Its lysosomotropic potential has been described as comparable to chloroquine [210]. Umifenovir is registered in Russia and China for the treatment of influenza [211]. It is claimed to inhibit the membrane fusion of viruses with the endosomal membrane, which possibly occurs via increasing endosomal pH [212]. An open-label, randomised, clinical trial in Iran has reported umifenovir significantly contributes to improvements in peripheral oxygen saturation and duration of hospitalisation for COVID-19 patients [213]. Therefore, while lysosomotropicity is a magic bullet pharmacological property against some parasites, it is a broad-spectrum magic shield property against viruses.

Correlation of CAD-induced phospholipidosis with antiviral activity only accounts for part of the anti-infective pharmacology of a subset of IAs; while tetracyclines are not CADs, the immunomodulatory activity of macrolides appears to be linked with phospholipidosis. In LPS-stimulated J774A.1 ​cells, the extent of inhibition of pro-inflammatory markers IL-6 and PGE2 by macrolides significantly correlated with their extent of accumulation in cells, as well as with induction of phospholipidosis [214]. While recent reviews are debating on whether phospholipidosis is a true antiviral property or merely an *in vitro* artefact, phospholipidosis as a host-targeted mechanism common to different antimicrobial classes has potential to be a foundational principle of network PPP [215,216].

### 3.2 Therapeutic range

As drug repositioning emerges as a cogent strategy for drug discovery, several studies will seek to delineate common properties of multi-purpose therapeutics (MPTs). MPTs can be defined as therapeutics which are repositioned according to more than one therapeutic class (analgesic, anticoagulant, anticancer, antidepressant, antidiabetic, antiepileptic, antimicrobial, antipsychotic, antipsychotic, antispasmodic, cardiovascular, depressant, sedative, stimulant). While many of these therapeutics are outside infectious disease research, the success of magic shields for treating infectious disease epitomises the need to include such host-directed drugs in antimicrobial research (Table 1).

**Table 1:** **Indications of MPTs.** As underlying host-directed pharmacology of MPTs may share similarities with IA polypharmacology so MPTs listed here are potentially hitherto uncharacterised IAs suitable for infectious disease.

Therapeutic	Original indication	Repositioned indication	Year	Pharmaceutical company
Amphotericin B	Fungal infection	Leishmaniasis	1997	NeXstar Pharma
Aspirin	Inflammation, pain	Antiplatelet, heart attack, stroke	Various	Various
Bromocriptine	Parkinson's disease	Diabetes mellitus	2009	Novartis
Bupropion	Depression	Smoking cessation	1997	GSK
Colchicine	Gout	Recurrent pericarditis	2009	URL Pharma
Duloxetine	Depression	Stress urinary incontinence; fibromyalgia; chronic musculoskeletal pain	2004; 2008; 2010	Lilly; Lilly; Lilly
Eflornithine	Cancer	African sleeping sickness	1990	Aventis
Everolimus	Immunosuppressant	Cancer	Various	Novartis
Finasteride	Hypertension	Male pattern baldness	1997	Merck
Gabapentin	Epilepsy	Neuropathic pain	2004	Parke Davis
Methotrexate	Cancer	Psoriasis, rheumatoid arthritis	2001	Barr Labs
Miltefosine	Cancer	Visceral leishmaniasis	2014	Zentaris
Minoxidil	Hypertension	Male pattern baldness	1988	Upjohn
Propranolol	Hypertension	Migraine prophylaxis, tremors	Various	Various
Sildenafil	Angina	Erectile dysfunction	1998	Pfizer
Zidovudine	Cancer	HIV/AIDS	1987	Burroughs

**Fig. 2:**
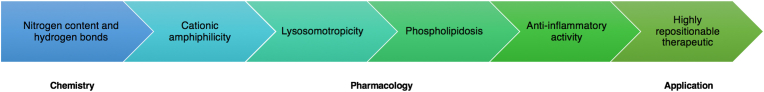
**Properties of MPTs.** Prospective properties range from pKa to polarisation of macrophages.

One insightful study has revealed that MPTs are hallmarked by a higher hydrogen-bond donor count, suggesting a greater number of hydrogen bonds could contribute to the pluripotent drug-target binding mechanism across multiple disease phenotypes [217]. It is conceivable that lysosomotropic drugs with an optimum number of hydrogen bonds, by globally shifting cellular homeostasis away from the disease state (i.e. shifting the damage-response curve downwards), may be used for both communicable and non-communicable diseases without contributing to AMR (Fig. 2). Therapeutics of the future may be furcated into acute and chronic classifications, where an indication is decided according to biomarker profiles across pathophysiology.

In conjunction with lysosomotropicity, disruption of mitochondrial function also appears to be a common denominator for MPTs, with psychotropic drugs, for example, showing anticancer activity by disrupting both mitochondrial and lysosomal function [218]. Similarly, antibiotics that target mitochondria effectively eradicate cancer stem cells across multiple tumour types. Mitochondrial biogenesis has been described as a global phenotypic characteristic highly conserved among cancer stem cells, enabling a mutation-independent approach to cancer therapy. 5 different classes of FDA-approved mitochondrially-targeted antibiotics include the erythromycins, tetracyclines, glycylcyclines, pyrvinium pamoate (an antiparasitic), and chloramphenicol. It has therefore been proposed that cancer can be treated like an infectious disease by repositioning FDA-approved antibiotics as preventative measures for anticancer therapy via eradication of cancer stem cells [219].

In closing, it is hoped that antimicrobial drugs of the future will be host-directed treatments that employ pharmacological mechanisms that ameliorate a range of non-communicable diseases, do not contribute to AMR, and ultimately antiquate traditional pathogen-killing antimicrobials.

## 4 IA development

Although IAs can be synthesised via traditional chemical and *in silico* methods of drug discovery (as with all small-molecule drugs), their PPP is only validated by repositioning. Following this, development of IAs for pandemics must involve 1) reducing their contributions to AMR and 2) increasing their therapeutic range. These can be achieved by chemical modification and validated by repositioning, both of which are accelerated by ever-evolving data-mining and machine learning (ML) processes which appraise chemical, pharmacological, and repositioning databases [220].

### 4.1 Chemical modification of existing antimicrobials

All small-molecule drugs used for infectious disease exhibit a conjectural bullet:shield ratio (BSR) which describes their pathogen-directed and host-modulating pharmacology. Reducing the bullet character of a given IA reduces its contributions to AMR [221]. Many IAs described in this review have already seen reduced BSRs by chemical modification; the archetype is the synthesis of non-antibiotic macrolides. In 2017, an azithromycin-based compound was found to reduce airway inflammation in chronic lung diseases whilst harbouring significantly diminished antimicrobial activity against a range of bacterial strains [222]. Moreover, the non-antibiotic macrolide EM900 inhibits rhinovirus infection and cytokine production in human airway epithelial cells [223].

Conjugation has also endowed existing antimicrobials with immunomodulatory activity. Influenza therapy with a single targeted compound is often limited in efficacy inasmuch as it contributes to AMR. However, uncontrolled release of virus-induced cytokines may contribute to high mortality of humans infected by H5N1 avian influenza virus. Therefore, novel dual-targeted bifunctional anti-influenza drugs were formed by conjugation with anti-inflammatory agents. The caffeic acid (CA)-bearing zanamivir (ZA) conjugates, ZA-7-CA (1) and ZA-7-CA-amide (7), showed simultaneous inhibition of influenza virus neuraminidase and suppression of pro-inflammatory cytokines [224]. These ZA conjugates provided protection of cells and mice against influenza infections. In fact, intranasal administration of low dosage (<1.2 ​μmol/kg/day) ZA conjugate exhibited much greater effect than the combination therapy with ZA and anti-inflammatory agents in protecting lethally-infected mice from H1N1 or N5N1 influenza viral infection. Conjugation to reduce BSR is a consolidative and promising area of research [225].

The BSR denotation for a given therapeutic is also dependent on the type of pathogen in consideration. The antibiotic azithromycin is a magic bullet for bacteria and malaria, but it is yet to be seen whether it behaves as a bullet across all parasite types; and azithromycin's *in vitro* antiviral activity emerges from its host-directed lysosomotropicity [226]. Bullet:shield variability therefore exists across pathogen types and perhaps within a pathogen class [227]. Nevertheless, ‘shielding’, or chemical modification of IAs as a way to reduce BSR, can yield non-AMR-contributing therapeutics.

### 4.2 Repositioning existing antimicrobials and non-antimicrobials

While chemical modification changes the real BSR of a therapeutic, repositioning changes its perceived BSR. As an example, repositioning of the macrolide antiparasitic ivermectin for COVID-19 has unearthed its antiviral applicability and has raised questions as to how much of its antiparasitic pharmacology is determined by its pathogen-directed properties [228,229].

Although repositioning of antimicrobials for non-communicable disease states have contributed to the acknowledgement of IAs, repositioning of non-antimicrobial drug classes towards infectious disease has also consolidated the potential of magic shields within the pathogenic context. Such shields have emerged from a variety of sources, from proton-pump inhibitors to antineoplastic agents [230]. Soon, antimicrobials that are crippled by their contributions to AMR, including IAs, may be replaced with those that exclusively exert network pharmacological effects on the host to achieve the same desired clinical outcome [231].

The molecular structures described in this review provide a starting point for drug-centric repositioning programmes which seek to discover non-AMR-inducing antimicrobials. Although a product of serendipity, there are 3 systematic approaches to drug repositioning: disease-centric, target-centric, and drug-centric [232]. Disease-centric approaches identify close relationships between an old and a new indication. A target-centric approach links a known target and its established drug to a new indication. Finally, a drug-centric approach connects a known drug to a new target and its associated indication. Despite the use of the umbrella term ‘drug’ repositioning, disease- and target-centric approaches have dominated the field; it has been suggested that the use of drug-centric approaches and structure-based techniques be exploited to realise the full potential of drug-target-disease connections. Analysis of repositioned drugs in an RDD database has revealed the majority of drugs have been redirected to the same disease family. This tendency was particularly pronounced within 2 categories of therapeutic indications: neoplasms and immune system disorders. These categories also have the highest number of repositioning cases in the database. Rapid repositioning approaches within the same disease family have been prioritised, leaving a pool of drug-target-disease connections unexplored [233]. The pleiotropic mechanism of action of IAs requires that IA-target-disease interactions be considered in the presence of a range of pathogen types.

While BSRs are a useful conceptual tool for understanding therapeutic contributions to AMR, they are confounded by the observation of the differential effect of a given therapeutic depending on its pathogen context. Azithromycin, for example, exhibits an altered immunomodulatory pharmacological landscape in the presence of pathogenic bacteria compared to viruses, furthering the need to consider a dynamic host-pathogen interactome model when screening therapeutic candidates and the need to recognise the importance of repositioning as a method for expanding our understanding of drug-disease networks [234].

## 5 Discussion

The term ‘IA’ sequesters a set of chemical compounds which harbour a pharmacological profile that is both unique and timely. The PPP of these compounds, which has not emerged from traditional magic bullet antimicrobial development and yet satisfies the need for host-directed, broad-spectrum antivirals, makes such therapeutics suitable for bioterrorism- and pandemic-preparedness research [235]. This broad pharmacology, likely emanating from their pKa and ability to alter intracellular pH, has also given rise to their applicability outside of infectious disease [236]. Indeed, by bridging the gap between unrelated disease types, IAs preface a therapeutic development paradigm beyond conventional nosology.

The rediscovery of existing antibiotics as anti-inflammatory agents is partly due to classical pharmacological approaches that have preceded modern target-based research. Classical pharmacology relies on phenotypic screening of chemical libraries of synthetic small molecules, natural products or extracts to identify substances that have a desirable therapeutic effect. The potency, selectivity, and other properties of these screening hits are optimised to produce candidate drugs. Only after the compounds have been discovered is an effort made to determine the biological target of the compounds through target validation experiments, often involving chemoproteomics. Since the 1990s, it has become popular to develop a hypothesis that a certain biological target is disease modifying and screen for compounds that modulate the activity of this purified target. Afterwards, these compounds are tested in animals to see if they have the desired effect; an approach known as ‘reverse pharmacology’ or target-based drug discovery [237]. However, recent statistical analyses revealed that a disproportionate number of first-in-class drugs with novel mechanisms of action emerge from phenotypic screening (classical pharmacology). The tendency to overestimate the ability of advances in basic research and brute-force screening methods to show a molecule as safe and effective in clinical trials has been described as a contributing factor to ‘Eroom's law’ in drug discovery [238]. While this review does not present an argument for either approach, it is important to consider many IAs such as ivermectin have been discovered through phenotypic screening and use in animals prior to humans [239].

The identification of chemical and pharmacological commonalities across classes of IAs has significant implications for antimicrobial development. One emergent theme is lysosomotropicity, a property likely underpinning the concerted way in which IAs alter cellular homeostasis prior to and/or during infection [240,241]. Last year, it was found that lysosomotropic agents including azithromycin, chloroquine, and hydroxychloroquine, can trigger the integrated stress response both *in vitro* and *in* *vivo* [242]. A link between lysosomotropicity and activation of the integrated stress response is perhaps one of many conserved, global mechanisms by which IAs achieve their PPP [243]. Further research will elucidate the links between lysosomotropicity and downstream network pharmacology.

The combinatorial use of IAs is another interesting avenue for further research. In one study over 20 years ago, nitazoxanide's treatment of cryptosporidiosis, a parasitic infectious disease, was evaluated in an *in vitro* system in combination with azithromycin and rifabutin [244]. Good *in vitro* activity of the drug combinations was observed with safe cytotoxicity levels. In the ensuing years, increasing global significance of cryptosporidiosis among children has renewed efforts to improve control measures [245]. A gnotobiotic piglet model of acute diarrhoea was used to probe azithromycin/nitazoxanide against *Cryptosporidium hominis*, the species responsible for most human cases [246]. 10 day-treatment with recommended doses for children showed that treatment with azithromycin or nitazoxanide relieved symptoms early compared with untreated animals, while treatment with both azithromycin and nitazoxanide led to considerable symptomatic improvement and modest reduction of mucosal injury. More recently, Kelleni's advocacy of azithromycin/nitazoxanide for COVID-19 has received significant academic attention [247,248]. Yet, there are currently no studies investigating azithromycin/nitazoxanide for respiratory diseases. Combinations of IAs with magic shields, such as the combination of azithromycin and glucocorticoids for COVID-19, is a parallel line of enquiry for future research [249].

In June 2022, the World Health Organization declared the monkeypox outbreak an ‘evolving health threat’. While not a Public Health Emergency of International Concern (PHEIC), the most significant viral outbreak since COVID-19 warrants an investigation into its IA candidates. In this regard, salicylanilides have received particular attention since May of this year with South Korea's Hyundai Bioscience's announcement to submit a fast-track processing request to the FDA for the drug CP-COV03, whose active ingredient is niclosamide [250]. This followed research from Kansas State University which reported that niclosamide reduced vaccinia virus (another orthopoxvirus) proliferation to 1% at a concentration as low as 1 μM [251]. Hyundai Bioscience CEO Oh Sang Ki stated ‘CP-COV03 is a universal antiviral drug with niclosamide as the main ingredient, which can fight nearly all types of viruses. If CP-COV03 is approved as a treatment for monkeypox with the FDA's fast-track designation, we will witness the birth of another innovative antiviral drug comparable to penicillin: the epitome of the 20th century's wonder antibiotics.’ In December 2021, the company reported that CP-COV03 plus dexamethasone had been effective against severe COVID-19 patients, another clinical success for an IA/magic shield combinatory treatment regimen [252]. In 2018, the salicylanilide nitazoxanide was found to inhibit vaccinia virus replication at a viral lifecycle stage after entry but before late gene expression [253]. It has been hypothesised that this mechanism is likely due to nitazoxanide's ability to interfere with metabolic adaptations needed for efficient virus replication, possibly by affecting acetyl-CoA production. The viruses now known to be sensitive to nitazoxanide include DNA and RNA viruses, viruses that replicate in the nucleus and in the cytoplasm, and enveloped and non-enveloped viruses. The extant application of traditionally antiparasitic therapeutics for monkeypox reaffirms the post-2020 epistemological shift from traditional magic bullet antivirals to host-modulating pan-pathogen pharmacological agents.

IAs are not the only class of antimicrobials that have challenged the magic bullet: magic shields can also be used to treat infectious disease, challenging previous denotations of the term ‘antimicrobial’. Decades of clinical investigations by van de Beek have cemented the administration of the anti-inflammatory agent dexamethasone for bacterial meningitis [254,255]. During the COVID-19 pandemic, the same glucocorticoid was discovered to treat severe illness by reducing hyperinflammation [256]. The applicability of dexamethasone for both bacterial and viral diseases in the clinical setting is evidence of the pan-pathogen success of host-directed small-molecule therapy and warrants further repositioning studies to truly elucidate dexamethasone's pan-pathogen potential. Unsurprisingly, as with IAs, dexamethasone is also used to treat cancers, including leukaemia and lymphoma [257]. It is important, therefore, to highlight both the pan-pathogen potential (and therefore pandemic-preparedness potential) and MPT potential of IAs and pure magic shield immunomodulators [258,259].

**Fig. 3:**
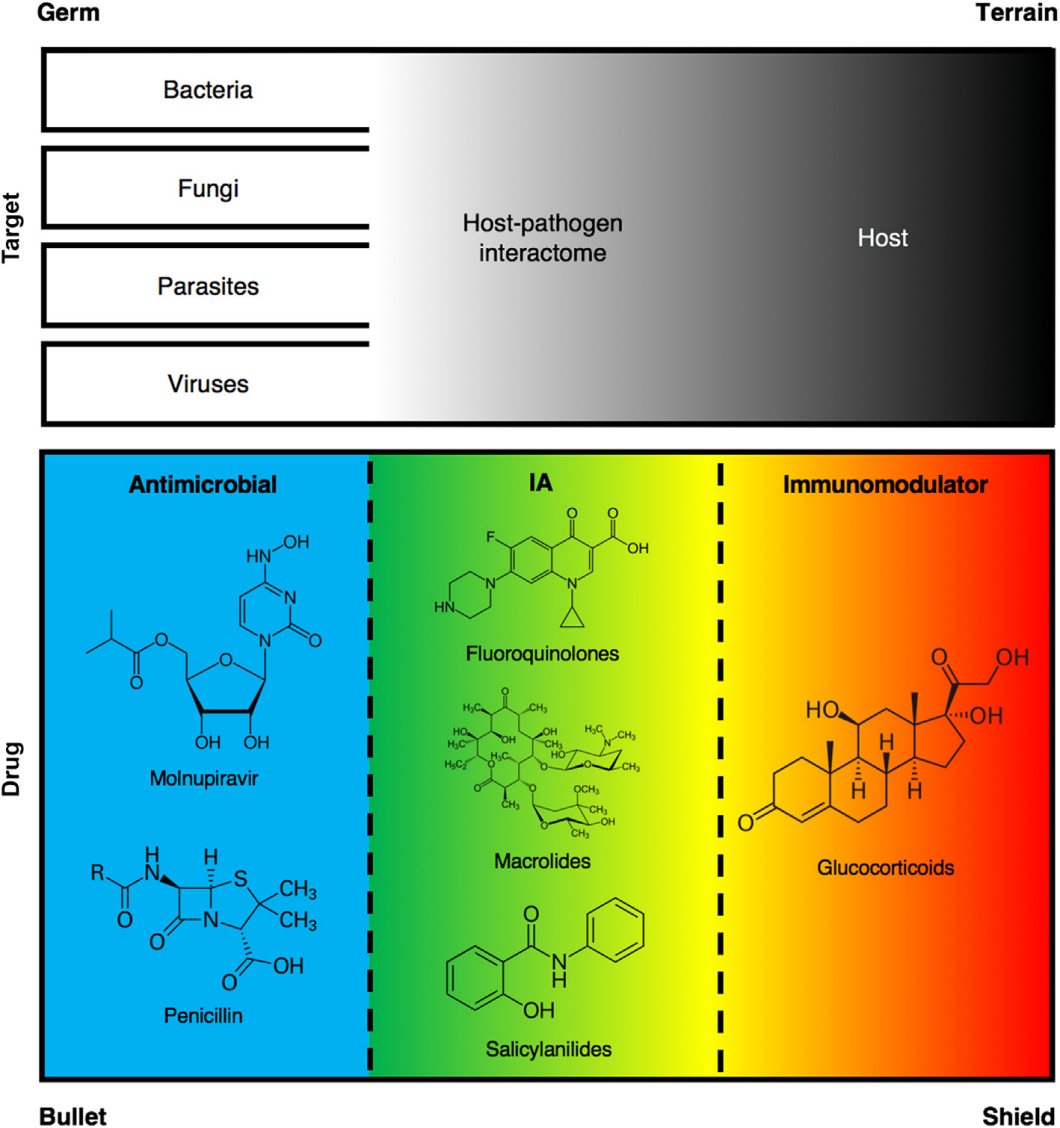
**Unification of four pillars of modern medicine: Pasteur's germ theory, Béchamp's terrain theory, Ehrlich's magic bullet, and the magic shield.** The magic bullet and the magic shield are not disparate concepts but two extremes of a continuum. All small-molecule drugs used to treat infectious diseases can be classified into 3 groups: antimicrobials, immunomodulators, and immunomodulating antimicrobials (IAs). This new model overcomes limitations of the magic bullet by recognising the polypharmacology of modern therapeutics and polymodality of the host target (indicated by gradients). As a result, the model predicts that A) Paragon magic shield immunomodulators such as dexamethasone can be used to treat infectious diseases such as COVID-19 and B) Both IAs and magic shields can be used against more than one pathogen class, giving rise to the phenomenon of pan-pathogen pharmacology (PPP). PPP is therefore a consequence of non-magic bullet antimicrobial development. Dotted lines indicate potential for shielding.

Ultimately, this review propounds a pandemic-preparedness drug development paradigm centred around the phenomenon of PPP. It can be argued that there are two types of PPP: the first is magic bullet PPP, whereby a drug exhibits the ability to directly inhibit multiple pathogen classes by targeting conserved moieties within pathogen morphologies. This has been achieved by non-small-molecule therapeutic avenues, such as silver nanoparticles. However, such technologies are yet to be trialled clinically and are still far from being an affordable intervention for global health emergencies [260]. The second, magic shield PPP, is the ability to modulate the host immune system in order to benefit the host during any type of infection across multiple pathogen classes and is displayed by all immunomodulators and IAs. Although some IAs display magic bullet PPP, I maintain that all derive their PPP from their network pharmacological, immunomodulating properties (Fig. 3) [[261], [262]].

## 6 Conclusion

By the time a vaccine had been developed, over 200,000 people had died from COVID-19: a grim reminder of the need for therapeutic intervention for novel infectious diseases. Yet, more than antivirals, it was immunomodulators like dexamethasone which displayed clinical effectiveness against COVID-19. Therefore, while the magic shield model is an epistemological departure from a century-spanning paradigm of modern medicine, it recognises the pan-pathogen potential of IAs and immunomodulators, continually reinforced by coetaneous repositioning.

## Availability of data and material

Not applicable.

## Author contribution statement

Praveen Prathapan conceived, wrote, and edited the manuscript.

## Code availability

Not applicable.

## Ethics approval

Not applicable.

## Funding statement

This research received no funding.

## Declaration of competing interest

The author declare no known competing financial interests or personal relationships that could have appeared to influence the work reported in this paper.
